# CDI Exerts Anti-Tumor Effects by Blocking the FoxM1-DNA Interaction

**DOI:** 10.3390/biomedicines10071671

**Published:** 2022-07-11

**Authors:** Woo Dae Jang, Mi Young Lee, Jihye Mun, Gyutae Lim, Kwang-Seok Oh

**Affiliations:** 1 Data Convergence Drug Research Center, Korea Research Institute of Chemical Technology, 141 Gajeong-ro, Yuseong-gu, Daejeon 34114, Korea; wdjang@krict.re.kr (W.D.J.); bflower2@krict.re.kr (M.Y.L.); jhmun@krict.re.kr (J.M.); gyutae@krict.re.kr (G.L.); 2 Department of Medicinal and Pharmaceutical Chemistry, University of Science and Technology, 176 Gajeong-ro, Yuseong-gu, Daejeon 34129, Korea

**Keywords:** CDI, FoxM1, FoxM1-DNA interaction, anti-tumor activity, RNA-Seq, molecular modeling

## Abstract

The Forkhead box protein M1 (FoxM1) is an appealing target for anti-cancer therapeutics as this cell proliferation-associated transcription factor is overexpressed in most human cancers. FoxM1 is involved in tumor invasion, angiogenesis, and metastasis. To discover novel inhibitors that disrupt the FoxM1-DNA interaction, we identified CDI, a small molecule that inhibits the FoxM1–DNA interaction. CDI was identified through an assay based on the time-resolved fluorescence energy transfer response of a labeled consensus oligonucleotide that was bound to a recombinant FoxM1-dsDNA binding domain (FoxM1-DBD) protein and exhibited potent inhibitory activity against FoxM1-DNA interaction. CDI suppressed cell proliferation and induced apoptosis in MDA-MB-231 cells obtained from a breast cancer patient. Furthermore, it decreased not only the mRNA and protein expression of FoxM1 but also that of downstream targets such as CDC25b. Additionally, global transcript profiling of MDA-MB-231 cells by RNA-Seq showed that CDI decreases the expression of FoxM1-regulated genes. The docking and MD simulation results indicated that CDI likely binds to the DNA interaction site of FoxM1-DBD and inhibits the function of FoxM1-DBD. These results of CDI being a possible effective inhibitor of FoxM1-DNA interaction will encourage its usage in pharmaceutical applications.

## 1. Introduction

The Forkhead box M1 (FoxM1) is one of the most frequently overexpressed transcription factors in many malignant tumors. FoxM1 belongs to the Forkhead Box family, which has a conserved DNA-binding domain responsible for binding of Fox proteins to consensus sites [1]. Crucially, FoxM1 is expressed in proliferating cells where it upregulates the expression of several genes associated with cell cycle progression and mitosis, such as cyclin B, survivin, aurora B kinase, Cdc25b phosphatase, and polo-like kinase 1 (Plk1) [2]. FoxM1 is an extensively studied member of the Forkhead Box family and is considered a pharmaceutical target for treating many malignant tumors since the discovery of a linkage between the activation of FoxM1 and oncogenes, such as members of the Ras-MAPK and sonic hedgehog pathways [3]. FoxM1 is upregulated in various carcinomas [4]. Additionally, FoxM1 primarily participates in tumorigenesis through transcriptional regulation of target genes involved in cancer initiation, progression, metastasis, invasion and drug resistance [5,6].

The FoxM1 expression is turned off in terminally differentiated cells [4]. In agreement, several studies in animal models with conditional deletion of FoxM1 or RNAi-mediated FoxM1 downregulation reported that inhibition of FoxM1 caused a striking reduction in the number and size of tumors [7,8,9]. Thus, FoxM1 is considered a prognostic factor and a promising candidate target for treating various cancers such as lung [10], breast [11], and prostate [12] cancers. However, despite this huge therapeutic potential, few studies have reported about FoxM1 inhibitor to modulate the activity and the transcriptional expression level of FoxM1 and related downstream genes during cancer development. Previous studies reported direct FoxM1 inhibitors that block binding to the DNA bases, including FDI-6 [13,14], honokiol [15], thiostrepton [16], and sinomycin A [17]. Natural compounds, such as honokiol, thiostrepton, and sinomycin A, are promiscuous molecules with potent off-target effects [18,19,20]. Thus, multi-targeting natural products cannot be used to elucidate the primary effects caused by the inhibition of FoxM1 binding to DNA. Meanwhile, FDI-6 selectively inhibits FoxM1 binding to genomic targets [13]. However, intracellular FoxM1 protein level was minimally affected by FDI-6 treatment on breast cancer cells [13,21]. Therefore, there is an urgent need to develop potent and selective drugs suppressing FoxM1 pathway with a clear mode of action.

In this study, we have identified (4aR,4bS,6aR,7R,10aR,10bR,12R,12aR)-3-Chloro-4,4a,5,6,6a,7,8,9,10,10a,10b,11,12,12a-tetradecahydro-7,10a-dimethyl-13-(1-methylethyl)-1, 4-dioxo-1H-4b,12-ethenochrysene-7-carboxylic acid (CDI) as a novel inhibitor of the FoxM1–DNA interaction. However, (4aR,4bS,6aR,7R,10aR,10bR,12R,12aR)-13-isopropyl-7,10a-dimethyl-1,4-dioxo-4,4a,5,6,6a,7,8,9,10,10a,10b,11,12,12a-tetradecahydro-1H-4b,12-ethenochrysene-7-carboxylic acid (Quinopimaric) and (3aR,3bS,5aR,6R,9aR,9bR,11R,11aR)-12-isopropyl-6,9a-dimethyl-1,3-dioxo-3,3a,4,5,5a,6,7,8,9,9a,9b,10,11,11a-tetradecahydro-1H-3b,11-ethenophenanthro [1,2-c]furan-6-carboxylic acid (Maleopimaric), which are CDI derivatives, have been reported to exhibit a broad spectrum of cytotoxic activity against non-small cell lung cancer, colon cancer, breast cancer, renal cancer, and leukemia [22]. The anti-cancer effect of CDI itself has not been previously reported. Therefore, we evaluated the anti-tumor activity of CDI and investigated whether it suppressed the transcription of genes under FoxM1 control.

## 2. Materials and Methods

### 2.1. Reagents and Antibodies

CDI (CAT#, NO75-0015) was obtained from ChemDiv (San Diego, CA, USA). The dsDNA binding domain of FoxM1 (FoxM1-DBD) tagged with His×6 was supplied by Dr. Shin SJ (Yonsei University College of Medicine, Seoul, Korea). Streptavidin-XL-665 and anti-His×6 cryptate were purchased from Cisbio (Codolet, France). Biotinylated oligomers (sense: 5′-Biotin-TEG-AAA CAA ACA AAC AAT C-3′ and antisense: 5′-GAT TGT TTG TTT GTT T-3′) were obtained from Bioneer (Daejeon, South Korea). FDI-6, which was used as a reference, and all other reagents, including MgCl_2_, EDTA, glycerol, DTT, NaCl, and Brij-35, were obtained from Sigma-Aldrich (St. Louis, MO, USA).

### 2.2. Time-Resolved Fluorescence Energy Transfer-Based FoxM1-DBD Binding Assay

A time-resolved fluorescence energy transfer (TR-FRET)-based FoxM1-DBD binding assay determined the inhibitory activity of CDI on FoxM1-DNA interaction. Briefly, to evaluate the % inhibition and IC_50_ value of CDI, a TR-FRET-based FoxM1-DBD binding assay was performed with His×6-tagged FoxM1-DBD, biotinylated dsDNA, and CDI for 30 min at 25 °C in binding buffer, supplemented with 1 mM MgCl_2_, 0.5 mM EDTA, 50 mM NaCl, 10 mM Tris-HCl (pH 7.5), 4% glycerol, and 0.5 mM DTT. Subsequently, it was incubated with a detection mixture containing streptavidin-XL665 and anti-His×6-cryptate for 1 h at 25 °C in detection buffer, comprising 50 mM HEPES pH 7.5, 200 mM NaCl, 5 mM EDTA, 0.02% Brij-35. The TR-FRET signals were measured using an Envision multimode plate reader (PerkinElmer Waltham, MA, USA) with TR-FRET readout options. The TR-FRET modes were set to a 100 μs delay time, 320 nm for excitation, and 665 and 615 nm for emission.

### 2.3. Cell Culture and Viability Assay

MDA-MB-231 was purchased from Korean Cell Line Bank and maintained at 1 × 10^6^ cells/mL in RPMI1640 supplemented with 300 mg/L L-glutamine, 25 mM HEPES, 25 mM NaHCO_3_, 10% fetal bovine serum, 100 IU/mL penicillin G, and 100 μg/mL streptomycin at 37 °C in a humidified atmosphere containing 5% CO_2_ and 95% ambient air. MDA-MB-231 cells were cultured at a density of 2 × 10^3^ cells/well into 96-well plates. Cell growth was followed for 72 h after stimulation in the presence or absence of CDI (1–30 μM). Medium alone represented the negative control. Cell proliferation was assessed in vitro using water-soluble tetrazolium salt WST-1 (Roche, Indianapolis, IN, USA).

### 2.4. Cell-Death Analysis

For cell-death analysis, MDA-MB-231 cells on 6-well plates (1 × 10^5^ cells/well) were treated with 2, 4, and 8 µM of CDI for 24 h. Apoptotic loss of membrane asymmetry was analyzed by triple staining with 10 µg/mL Hoechst 33342 (Thermo Fisher Scientific, Bartlesville, OK, USA), 1 µg/mL fluorescein isothiocyanate annexin V (BD Biosciences, San Jose, CA, USA), and 10 µg/mL propidium iodide (BD Biosciences). Quantitative analysis of apoptotic cells and necrotic cells was performed using the NucleoCounter^®^ NC3000TM (Chemometec, Allerod, Denmark). Dimethyl sulfoxide (DMSO)-treated cells were used as controls.

### 2.5. Real-Time Quantitative PCR Analysis

The mRNA expression levels of FoxM1c and FoxM1 downstream targets such as Cdc25 were determined by real-time quantitative PCR service in COSMO Genetech (Sungsoo, Seoul, Korea). Similar to the cell-death analysis, MDA-MB-231 cells were treated with 2, 4, and 8 µM of CDI for 24 h. Total RNA was extracted from cultured cells using a RNeasy mini kit (Qiagen, Valencia, CA, USA). Gene expression was measured by real-time quantitative PCR (RT-qPCR) using a Bio-Rad CFX96 system (Hercules, CA, USA). The primer sequence was performed in accordance with Chen et al. (2015) [23]. For FoxM1c, the forward primer was 5′-CAA TTG CCC GAG CAC TTG GAA TCA-3′, and the reverse primer was 5′-TCC TCA GCT AGC AGC ACC TTG-3′. For CDC25b, the forward primer was 5′-GTG CTT GGT CTG TTT GAC TTT AC-3′, and the reverse primer was 5′-GAC CGA GTG GGT AAC TGA TAT TT-3′. A housekeeping gene, Glyceraldehyde 3-phosphate dehydrogenase (GAPDH), was used as the internal control. The forward primer for GAPDH was 5′-ACC CAG AAG ACT GTG GAT GG-3′, and the reverse primer was 5′-TGC TGT AGC CAA ATT CGT TG-3′. The PCR conditions for all genes were 40 cycles consisting of pre-denaturation at 94 °C for 3 min, amplification at 62 °C for 40 s, and 94 °C for 10 s. The qPCR was analyzed using the CFX manager (Ver. 3.1). Each sample was analyzed four times, and each result was concluded from four independent experiments.

### 2.6. Western Blot Analysis

Whole cell extracts were prepared in RIPA buffer (Thermal Scientific, Pierce, Rockford, IL, USA). Protein lysate (30 μg) was electrophoresed in a 10% SDS-PAGE gel (Bio-Rad, Hercules, CA, USA) and was transferred to a nitrocellulose membrane (Hybond-C Extra; GE Healthcare, Piscataway, NJ, USA) overnight at 75 mA and 4 °C. The next day, the nitrocellulose membrane was blocked with 5% milk and incubated with 1:1000 primary antibodies (FoxM1 Rabbit-mAb, #5436, Cell Signaling Technology, Denver, MA, USA) overnight at 4 °C. The secondary antibodies (HRP-conjugated goat anti-rabbit IgG, #31462, Thermal Scientific, Rockford, IL, USA) were diluted to 1:3000 in a fresh blocking solution. Protein bands were detected using the Super Signal West Femto kit (Thermo Fisher Scientific Inc., Waltham, MA, USA).

### 2.7. RNA Isolation and Sequencing

Cells were treated with DMSO (0.1%) or CDI compound (8 μM, 6 h), and total RNA was extracted using TRIzol reagent. The cDNA libraries were prepared using the TruSeq RNA library protocol (Illumina, San Diego, CA, USA). The cDNA was amplified by PCR for the enrichment of adapter-ligated fragments. After checking the sample quality for sequencing, such as the size of PCR enriched fragments and quantification of prepared libraries, samples from each group were sequenced. Quality and quantity of the amplified cDNA library were checked by Macrogen (Seoul, Korea). For paired-end sequencing, the amplified products were loaded to Illumina NovaSeq (Illumina, San Diego, CA, USA). The raw reads generated were subjected to a quality check using FastQC v0.11.7 (http://www.bioinformatics.babraham.ac.uk/projects/fastqc, date of last access 8 July 2022), and the low-quality reads and adapter sequences were filtered using Trimmomatic (v0.38, Aachen, Germany) [24]. The filtered reads from all the samples were aligned to the reference human genome (GRCh38.p13; assembly accession, GCF_000001405.39) using HISAT2 (v2.1.0, Baltimore, Maryland, USA) [25]. The resulting alignment read counts and assemblies were generated using StringTie (v2.1.3b, Baltimore, Maryland, USA) [26].

### 2.8. RNA-Seq Transcriptional Profiling

Differential expression analysis was performed using DESeq2 (v1.36.0, Heidelberg, Germany) [27]. The triplicate CDI-treated samples were tested for differential expression against the control condition (DMSO treatment). For differential expression analysis, we considered only the genes with an absolute logarithm of fold change (log_2_FC) > 2 and *p*-value < 0.05. Enhanced Volcano was used to depict the volcano plot (https://github.com/kevinblighe/EnhancedVolcano, date of last access 8 July 2022).

Gene Set Enrichment Analysis (GSEA) was used to examine our genome-wide expression profiles [28]. REVIGO (Reduce and Visualize Gene Ontologies) was utilized to visualize overrepresented Gene Ontology (GO) biological processes [29].

### 2.9. Docking and Molecular Dynamics (MD) Simulations

The protein structure of FoxM1-DBD was downloaded from PDB (PDB ID: 3G73) [30]. The 3G73 is a dimeric structure including DNA, with CDI docked on the ‘A’ chain. The Simplified Molecular Input Line Entry System (SMILES) [31] of CDI was ‘CC(C)C1=CC23CCC4[C@](C)(CCC[C@@]4(C)C(O)=O)C2CC1C1C3C(=O)C(Cl)=CC1=O’, which was converted into a three-dimensional structure using MarvinSketch program (Marvin 21.8.0, 2021, ChemAxon (http://www.chemaxon.com)).

Autodock Vina (v1.1.2, California, USA) was used for docking simulations. The CDI docking positions were designated as N283, R286, and H287, the residues where FoxM1-DBD and DNA are known to interact. The Cα atom of each residue was used as the center of the docking space, and a cube-shaped docking box with a volume of 20 Å^3^ was formed. The docking simulation was repeated 10 times at each docking space using random seeds, and 20 docking poses were generated in each simulation. After the docking process, the docked structures with similar poses were clustered and classified using the CHARMM program [32]. Then, by applying Gibbs free energy (Δ*G* = lowest energy + (−*kT*ln*N*), where the lowest energy value is from docking energy, *kT* ≈ 0.6, and *N* is the number of similar ligand structures in cluster), structures with low docking energy and similar poses were preferentially selected.

MD simulation was performed using GROMACS (v2020.4, Stockholm, Sweden) [33]. The best docking simulation result was used as an initial structure, and the CHARMM36 force field was used as the default force field. The restraint of CDI compound was generated by CGenFF (http://cgenff.umaryland.edu, Baltimore, USA, date of last access 8 July 2022) [34]. A dodecahedron-type water box was formed in an explicit solvent system, and TIP3 was used for water molecules. Short minimization (100 ps), NVT, and NPT were performed for chemical equilibrium, and MD simulation was performed for 100 ns.

### 2.10. Statistical Analysis

All values are expressed as means ± SD. Data were analyzed using one-way analysis of variance (ANOVA), followed by Dunnett’s test for multiple comparisons (Sigma Stat, Jandel Co., San Rafael, CA, USA). Concentration-response curves were analyzed through nonlinear regression using PRISM version 3.0 (GraphPad Software Inc., La Jolla, CA, USA), and the IC_50_ value of kamolonol (the concentration required to reduce the TR-FRET count to 50% of the positive control) was calculated. In all comparisons, statistical significance was accepted for *p* values below 0.05.

## 3. Results

In previous studies, quinopimaric and maleopimaric acid have shown anti-tumor effects for various cancers [22,35]. We intended to identify novel compounds through chemical similarity using quinopimaric and maleopimaric acid as reference compounds. CDI was identified by screening natural product-derived libraries, including quinopimaric and maleopimaric acid derivatives, through a TR-FRET-based FoxM1 binding assay. Since CDI did not report any anti-cancer effect, it was decided to analyze the inhibitory effect on FoxM1-DBD.

### 3.1. Inhibitory Effects of CDI on FoxM1-DNA Interaction

**Figure 1:**
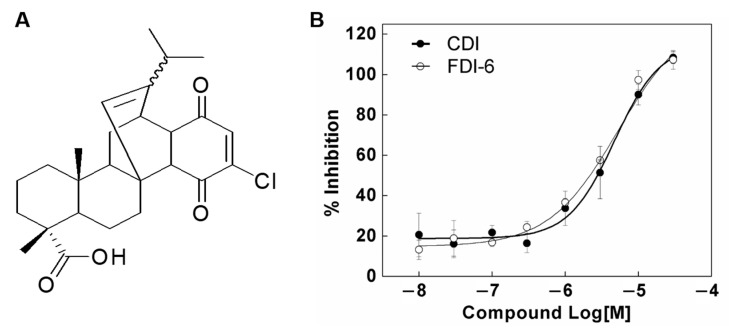
(**A**) Chemical structure of CDI and (**B**) the inhibitory effect of CDI shown with a TR-FRET-based FoxM1–DBD binding assay. The inhibitory responses were measured through a reaction with 200 nM FoxM1-DBD and 50 nM biotin-labeled oligomer DNA in the presence of a serial dilution of CDI and FDI-6 (10 nM–30 μM). Data are expressed as means ± SD (*n* = 3).

We performed several experiments to identify the inhibitory effect of CDI. First, the inhibitory effect of CDI on FoxM1–DNA interaction was evaluated using a TR-FRET-based FoxM1–DBD binding assay [36]. It involved specific interactions between the DBD of FoxM1 and tandem repeats dsDNA of consensus sequence (5′-AAA CAA ACA AAC AAT C-3′ and 5′-GAT TGT TTG TTT GTT T-3′), which is the known FoxM1 recognition motif on DNA. The quaternary complex of biotinylated dsDNA, a His×6-tagged FoxM1-DBD, a Europium (Eu) cryptate-labeled anti-His×6 monoclonal antibody, and streptavidin-XL665 can be directly quantified and expressed as TR-FRET counts, which reflect the amount of FoxM1–DNA interaction. The assay conditions of the TR-FRET-based FoxM1–DBD binding assay were verified using FDI-6 as a reference compound (IC_50_ values: 3.7 ± 0.9 µM). As shown in Figure 1, CDI inhibited the FoxM1–DNA interaction-induced TR-FRET counts in a concentration-dependent manner and provided IC_50_ values of 4.9 ± 1.5 µM.

### 3.2. The Effects of CDI on the Proliferation of MDA-MB-231 Breast Cancer Cell Line

**Figure 2:**
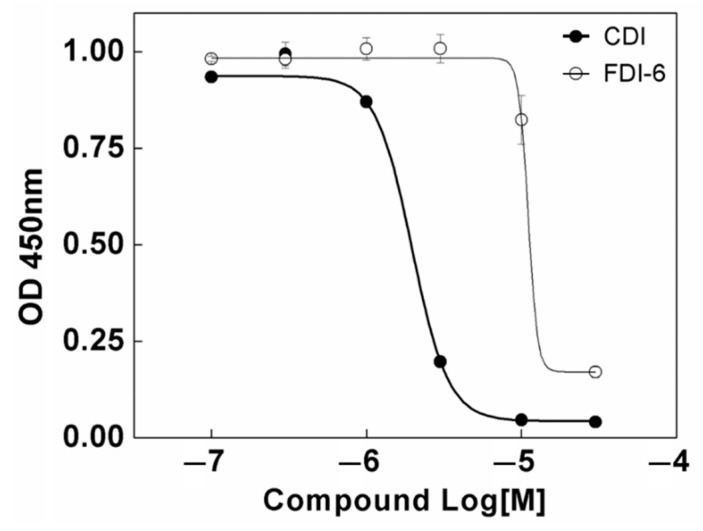
Anti-proliferative effects of CDI (●) and FDI-6 (◦) in MDA-MB-231 cell line. The MDA-MB-231 cells were incubated with 0.1–30 μM CDI and FDI-6 and were assayed at the 72-h time point using WST cell proliferation reagent. All data are presented as means ± S.D. of three independent experiments.

After it was confirmed that CDI directly interacts with FoxM1 to exhibit an inhibitory effect, the anti-proliferative effect in the tumor was confirmed. To evaluate the anti-proliferative effects of CDI, MDA-MB-231 cells, which highly express FoxM1, were treated with 0.1–30 μM CDI for 72 h. As shown in Figure 2, CDI inhibited the proliferation of MDA-MB-231 more efficiently (IC_50_ values: 4.1 ± 0.6 µM) compared to FDI-6 (IC_50_ values: 28.6.1 ± 0.4 µM).

### 3.3. The Effects of CDI on the Expression Levels of FoxM1c and FoxM1 Downstream Target

**Figure 3:**
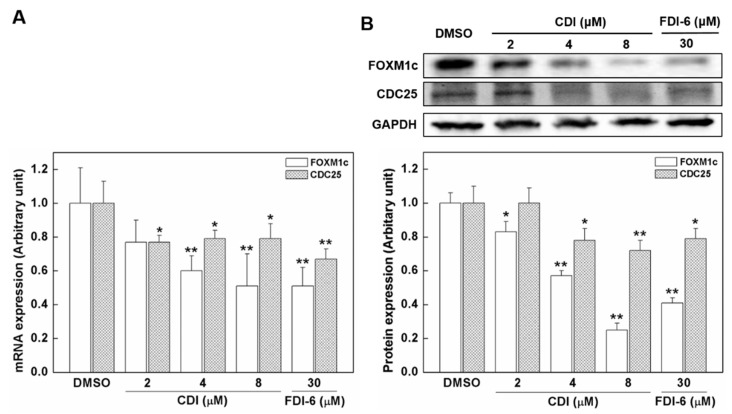
(**A**) The mRNA and (**B**) protein expression levels of FoxM1c and FoxM1 downstream targets, CDC25b, in MDA-MB-231 cells following CDI application. MDA-MB-231 cells were treated with 2–8 μM CDI and 30 μM FDI-6, and gene expression was measured by real-time quantitative PCR after 24 h. In addition, protein expression was measured by western blotting. Controls were treated with DMSO or FDI-6, and values are mean ± SD (*n* = 3). * *p* < 0.05 and ** *p* < 0.01 signifies which is significantly different from the DMSO control, respectively.

The inhibitory effects of CDI affect not only FoxM1 but also downstream targets. To confirm this, we investigated the mRNA and protein expression of the downstream target. The effect of CDI on mRNA expression of FoxM1c (auto-regulation) and FoxM1 downstream targets, namely CDC25b, was investigated in MDA-MB-231 cells. After 24 h of CDI exposure, FoxM1c was downregulated in MDA-MB-231 cells. The level of FoxM1c decreased in a concentration-dependent manner of CDI. In the downstream transcriptional analysis, the mRNA expression levels of CDC25 also decreased in accordance with the concentration of CDI (Figure 3A). Consistent with these results, the protein levels of FoxM1c and CDC25 also decreased according to the concentration of CDI (Figure 3B).

### 3.4. Apoptosis Induced by CDI in MDA-MB-231 Breast Cancer Cell Line

**Figure 4:**
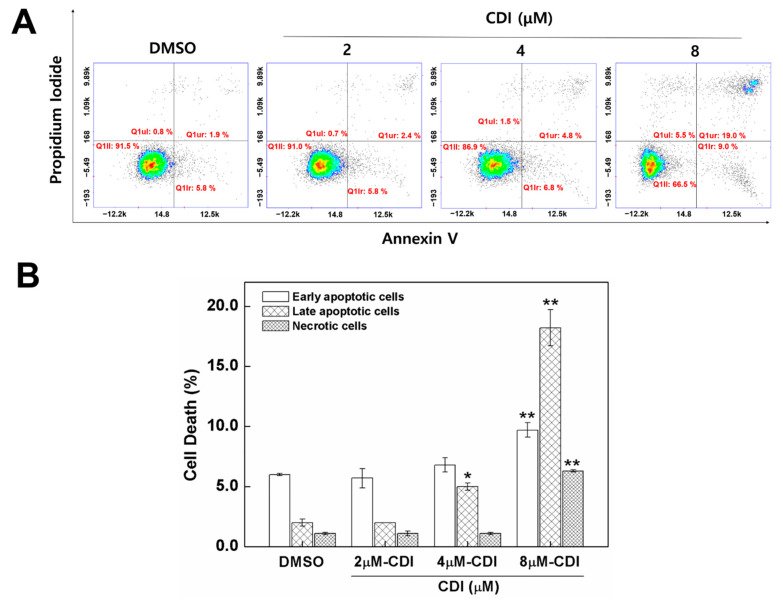
Effects of CDI on apoptosis and necrosis in MDA-MB-231 cell line. (**A**) MDA-MB-231 cells were treated with CDI concentrations of 2, 4, and 8 µM for 24 h. Apoptotic loss of membrane asymmetry was analyzed by annexin V and propidium iodide double-staining. (**B**) Quantitative analysis (*n* = 6) of apoptotic cells and necrotic cells using NucleoCounter^®^ NC3000TM (v2.1.25.12, Chemometec, Allerod, Denmark). DMSO-treated cells were used as control. Data are expressed as means ± SD (*n* = 3). * *p* < 0.05 and ** *p* < 0.01 signifies which is significantly different from the DMSO control, respectively.

FoxM1 inhibition causes cell growth inhibition and cell death [8]. Hence, to measure the impact of CDI on FoxM1-mediated apoptosis and necrosis, MDA-MB-231 cells were treated with DMSO and 2, 4, and 8 µM CDI for 24 h and stained with fluorescein isothiocyanate-annexin V and propidium iodide (PI), which are the indicators of apoptosis and necrosis. Using the NucleoCounter (v2.1.25.12, ChemoMetec, Allerod, Denmark), which performs cell counting based on the fluorescence, cells were classified as viable, early apoptotic, late apoptotic, or necrotic. As shown in Figure 4, CDI increased the apoptosis rate of MDA-MB-231 cells. Specifically, the percentage of late apoptosis increased from 2.0 ± 0.3% in DMSO groups to 2.0 ± 0.1%, 5.0 ± 0.3%, and 18.2 ± 1.5% in the presence of 2, 4, and 8 μM CDI, respectively. Similarly, the percentage of early apoptosis also increased from 6.0 ± 0.1% in the DMSO group to 5.7 ± 0.8%, 6.8 ± 0.6%, and 9.7 ± 0.6%, in the presence of 2, 4, and 8 μM CDI, respectively.

### 3.5. RNA-Seq Analysis of the Effects of CDI on Global FoxM1 Gene Regulation

**Figure 5:**
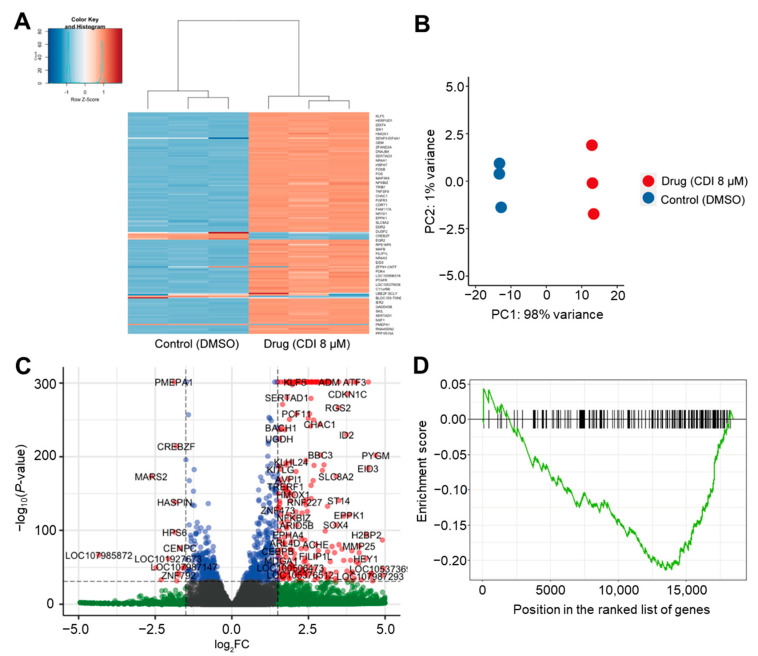
RNA-Seq analysis of MDA-MB-231 cells at the 6-h time point with 8 μM CDI or DMSO solvent. (**A**) Clustered heat map illustrating the normalized expression level of genes between control (DMSO) and compound treatment (CDI 8 μM) groups. (**B**) PCA analysis of gene expression profiles, showing the similarity between control (DMSO) and compound treatments (CDI 8 μM). (**C**) Volcano plot depicting the –log_10_ (*p*-values) across biological triplicates for the fragment per kilobase per million read fold changes. Each point represents a transcript mapped to the human genome. Significantly upregulated and downregulated transcripts are colored in red. The threshold for significance was set at *p*-value < 10^−32^ and log_2_ fold change at 1.5. (**D**) Enrichment plots of Gene Set Enrichment Analysis (GSEA) results. MDA-MB-231 cells treated with CDI (8 μM, 6 h). For GSEA analysis, gene sets of FoxM1 target genes were used that were derived from FoxM1 cistrome based on ChIP-seq experiments [37].

**Figure 6:**
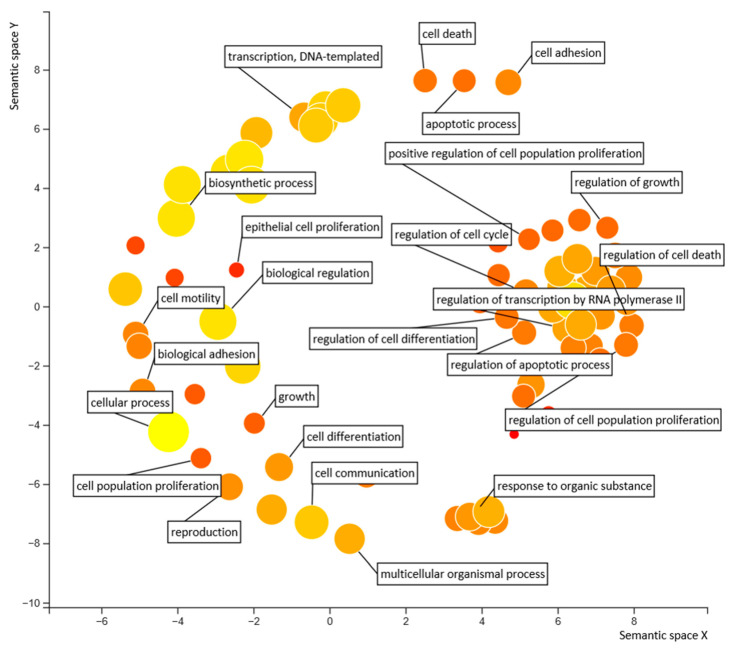
REVIGO (Reduce and Visualize Gene Ontologies) analysis of CDI. REVIGO reveals impacted Gene Ontology (GO) biological processes in CDI treated MDA-MB-231 cells. The circle sizes represent the number of genes in the GO term. Colors represent similarities along semantic space Y.

As FoxM1 is a transcriptional factor, we expected that the inhibition of FoxM1–DNA binding should lead to specific downregulation of target genes. Therefore, it was necessary to investigate the global transcriptional effects of CDI using RNA-Seq and GSEA. Considering the concentration-dependent effects of CDI on apoptosis and mRNA expression level (Figure 3 and Figure 4), we next examined the effect of CDI treatment (8 μM) on RNA transcript levels in MDA-MB-231 cells at 6 h. The experiment was performed in triplicate for the CDI treatment group and the untreated control. Clustering of gene expression profiles showed that independent replicates of the control (DMSO) and drug treatment (CDI, 8 μM) groups and their expression profiles exhibit robust reproducibility (Figure 5A,B). Figure 5C depicts differentially expressed genes (DEGs) upon CDI treatment (8 μM, 6 h) with upregulated and downregulated transcripts plotted by the logarithm of fold change (log_2_FC, *x*-axis) and the logarithm of the *p*-value (*y*-axis). Each point represents a transcript mapped to the human genome. Significantly upregulated and downregulated transcripts with log_2_FC at 1.5 and –log(*p*-value) > 32 are colored in red (Figure 5C). Of the transcripts changed by 1.5-fold or greater in CDI-treated cells, 260 transcripts were significantly upregulated, while 17 were significantly downregulated. We expected that CDI-induced downregulation of FoxM1-regulated genes, changes in secondary and tertiary downstream responses to the suppression of FoxM1, and non-FoxM1-specific gene expression would all respond to the stress of drug treatment. Importantly, differential gene expression data against gene sets consisting of FoxM1 target genes revealed negative enrichment scores by GSEA, indicating that CDI downregulates the expression of genes in the FoxM1 cistrome (Figure 5D). For GSEA analysis, gene sets of FoxM1 target genes were derived from FoxM1 cistromes based on ChIP-seq experiments [37]. The major categories of gene regulations were identified by GO analysis and shown in the reduce and visualize gene ontologies (REVIGO) [29] plots (Figure 6). REVIGO analysis shows that biological processes well known to be under FoxM1 regulation, such as cell cycle, apoptosis, and proliferation, are most affected by CDI-treated MDA-MB-231 cells.

### 3.6. Computational Simulation Results between FoxM1-DBD and CDI

**Figure 7:**
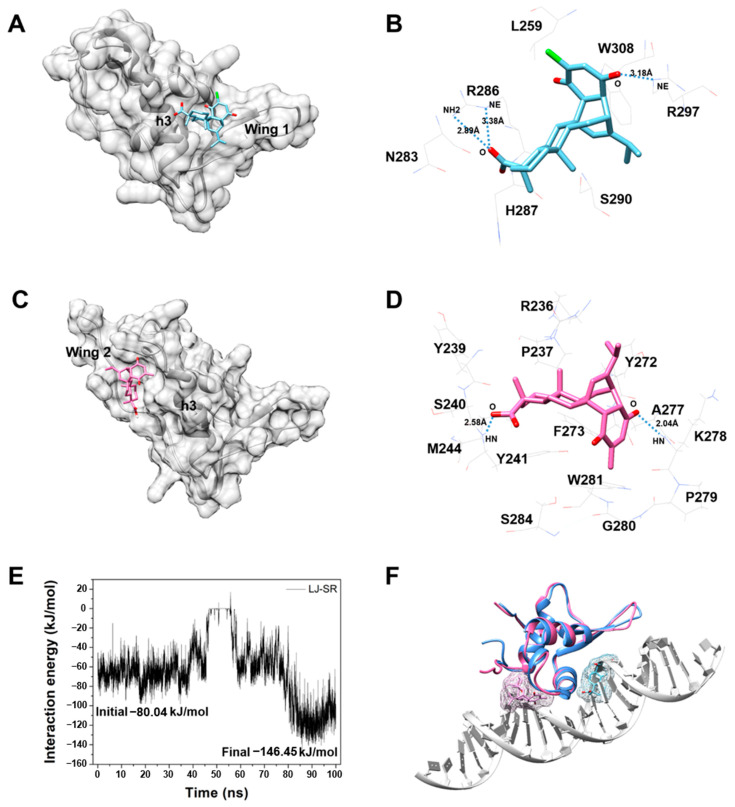
Computational simulation results involving interaction between CDI and FoxM1-DBD. (**A**) Docking result structure with the lowest energy. FoxM1-DBD is represented by the grey ribbon and surface, CDI is represented by the blue stick. (**B**) Interaction residues from docking results. Wire sticks are interacting residues. Dotted line indicates hydrogen bonds. (**C**) The structure from MD result. A grey ribbon and surface model is a FoxM1-DBD structure, and a pink stick represents CDI. (**D**) Interaction residues from MD results. Wire sticks are interacting residues. Dotted lines indicate hydrogen bonds. (**E**) Protein-ligand interaction energy profile obtained using 100 ns simulation. (**F**) Binding position comparison between docking and MD simulations. Blue structure is from the docking result and pink structure is from the MD result. Chimera program was used for drawing 3D structures [38].

We confirmed the inhibitory effect of CDI through experiments and gene analysis. Simulations were performed using computational methods to confirm a more specific inhibition mechanism. Docking and MD simulations were performed to investigate the inhibitory effect of CDI on FoxM1-DBD through its binding and movement. As a result of docking simulation, 600 binding poses capable of inhibiting the binding of FoxM1-DBD to DNA were obtained. Among them, the structure with the most stable docking energy was selected. The CDI with docking energy of −8.3 kcal/mol bound to FoxM1-DBD between the ‘Wing’ region and helix ‘h3′ (Figure 7A). CDI interacted closely with L259, N283, R286, H287, S290, R297, and W308 of FoxM1-DBD and particularly, formed strong hydrogen bonds on E286 and R297 (Figure 7B).

Based on the docking simulation results, MD simulations were performed to analyze the movement of CDI and to identify the possibility of new binding sites. The MD simulation can achieve better results because it can resolve the clash of atoms caused by fixed protein structure and does not consider the solvation system while docking simulations. Therefore, we performed a 100 ns MD simulation using the docking result as an initial structure to obtain a more accurate interaction.

As shown in Figure 7C, the position of the CDI that was bound to the initial structure moved to the opposite side based on the position of helix ‘h3′, which interacts directly with DNA. The protein-ligand interaction energy of the moved binding site was approximately 1.8 times more stable from –80.04 kJ/mol to –146.45 kJ/mol (Figure 7E). The moved CDI interacted with R236, P237, Y239, S240, Y241, M244, Y272, F273, A277, K278, P279, G280, W281, and S284, and formed hydrogen bonds with T241 and K287 (Figure 7D). These simulation results confirmed that CDI can bind to both sides based on the helix ‘h3′ of FoxM1-DBD (Figure 7F).

## 4. Discussion

CDI is a natural product-like compound with pimaric moiety. Although quinopimaric and maleopimaric acid have a broad spectrum of anti-tumor activities [22,35], little information is available about the pharmacological actions of CDI. In addition, we have continuously tried to discover FoxM1 inhibitors in the natural product library for the past several years. Compared to the existing compounds, CDI predicted from our system [39,40,41,42,43,44] has been analyzed to have an excellent overall pharmaco-profile and stability to various toxicities. Furthermore, in terms of efficacy, anti-tumor effects have not been reported. Therefore, in the present study, we have identified CDI that blocks FoxM1-DNA interaction using a FoxM1-DBD binding assay based on TR-FRET [36]. CDI showed potent ability to inhibit the binding of FoxM1 to target DNA. We characterized CDI at the transcriptome level to show that CDI broadly represses the transcription of FoxM1-regulated genes by inhibiting FoxM1 binding to DNA. Previous RNA-Seq studies reported that several FoxM1 inhibitors simultaneously downregulated multiple FoxM1-regulated genes [13,21]. Consistent with this, we found that most downregulated DEGs in CDI-treated MDA-MB-231 cells corresponded to known FoxM1 target genes based on ChIP-seq analysis. In addition, computational studies, docking, and MD simulations also suggested that CDI was stabilized at the DNA interaction site around helix ‘h3′ with hydrogen bonds and van der Waals interactions. This indicates that CDI can act as an inhibitor by physically blocking the binding between FoxM1-DBD and DNA.

Fox proteins are a superfamily of evolutionarily conserved transcriptional regulators defined by a common DBD termed the ‘Forkhead box’ or ‘winged helix’ domain. Fox proteins are essential components of oncogenic and tumor suppressive pathways [45]. Overexpression of FoxM1 promotes cell-cycle progression, leading to tumorigenesis and cancer progression. By contrast, FoxO3 may increase resistance to apoptosis and cell-cycle progression, and deregulation of FoxO3 can result in cancer progression [3]. To confirm the selectivity of CDI for FoxM1, we compared the expression of patterns of FoxM1 and FoxOs downstream target genes obtained through RNA-Seq data. The majority of the FoxM1 target genes were downregulated as a result of CDI treatment (Figure 5D). These include *CCNB1* (log_2_FC, −0.392), *CDC25A* (log_2_FC, −0.229), and *CENPF* (log_2_FC, −0.596). In particular, because FoxM1 is involved in a positive autoregulatory loop that induces its transcription [46], inhibition of FoxM1 by CDI leads to the downregulation of its mRNA level (log_2_FC, −0.177). On the other hand, no significant downregulation was observed for known downstream genes of FoxOs. For example, these include *CDKN1A* (log_2_FC, 2.433), *FASLG* (log_2_FC, 3.683), *BCL2L11* (log_2_FC, 1.165), and *GADD45A* (log_2_FC, 1.458). These results suggest that CDI exhibits pharmacological action by specifically binding to FoxM1, not FoxOs. In fact, despite the highly conserved DBD of Fox proteins, previous studies have already shown that it is possible to develop specific Fox inhibitors that the individual Fox-DBD interactions [13,47,48].

FoxM1 functions as a typical transcription factor which regulates a network of proliferation-associated genes critical to mitotic spindle assembly [49], chromosome segregation [50], and G2/M transition [51]. Furthermore, aberrant upregulation of FoxM1 has been implicated in tumorigenesis and tumor progression of breast cancer [8]. In the present study, the cytotoxic effect of CDI on breast cancer was examined by using a WST cell viability assay. The results confirmed that CDI alone was able to reduce the percentage of viable breast cancer cells by inhibiting FoxM1–DNA interaction. Previous studies have demonstrated that small molecule inhibitors of FoxM1 that block DNA binding, including FDI-6, thiostrepton, and sinomycin A, promote cancer cell death by apoptosis and by inducing cell cycle arrest [52,53,54,55]. Natural compounds, such as thiostrepton and sinomycin A, are promiscuous molecules with potent off-target effects [18,19,20]. Thus, these compounds cannot be used to elucidate the primary effects caused by the inhibition of FoxM1 binding to DNA. The synthetic compound, FDI-6, selectively inhibited FoxM1 binding to DNA, but intracellular FoxM1 protein levels were minimally affected by FDI-6 treatment on breast cancer cells. Meanwhile, CDI reduced the protein level of FoxM1 at a much lower concentration than that of FDI-6 (Figure 3B). Also, we have observed that CDI increased the apoptotic rate in a concentration-dependent manner.

While our study provides robust evidence of the potential of CDI as an anti-cancer molecule, all our experiments were done in breast cancer cell lines. Hence, future studies are required to test the results reported here in animal models of breast cell cancer as well as other cancers.

## Data Availability

Not applicable.
